# Comparative Efficacy of Microfluidics and Density Gradient Centrifugation for Sperm Preparation in IVF: A Randomized Controlled Trial

**DOI:** 10.5935/1518-0557.20250190

**Published:** 2026

**Authors:** Do Thuy Huong, Do Thi Minh Tam, Nguyen Thanh Hoa, Ho Nguyet Minh, Nguyen Manh Ha, Ho Sy Hung

**Affiliations:** 1 Hanoi Medical University, Hanoi, Vietnam; 2 Hanoi Medical University Hospital, Hanoi, Vietnam; 3 Faculty of Medicine, Nursing and Health Sciences, Monash University, Melbourne, Australia; 4 National Hospital of Obstetrics and Gynecology, Hanoi, Vietnam

**Keywords:** microfluidics, DNA fragmentation index, density gradient centrifugation, IVF, live birth

## Abstract

**Objective:**

To compare the effectiveness of microfluidic sperm sorting (MC) and traditional density gradient centrifugation (DGC) in reducing sperm DNA fragmentation index (DFI) and evaluate their impact on clinical outcomes in IVF cycles.

**Methods:**

In this randomized controlled trial, 119 couples undergoing IVF were allocated to either the MC or DGC group. Sperm DNA fragmentation was assessed before and after preparation. Primary outcomes included live birth rate and DFI reduction. Secondary outcomes were sperm quality parameters, fertilization rate, embryo development, and pregnancy outcomes. Subgroup analysis was conducted based on initial DFI levels (<15% vs. ≥15%).

**Results:**

The MC group demonstrated significantly greater and more consistent DFI reduction, particularly in samples with high baseline DFI (≥15%), compared to the DGC group. Although the number of oocytes retrieved was higher in the MC group, no significant differences were observed between groups in fertilization rate, Day-2 embryo quality, clinical pregnancy, ongoing pregnancy, or live birth rates. Subgroup analysis also showed no significant differences in outcomes based on baseline DFI levels.

**Conclusions:**

Microfluidics is an effective method for reducing sperm DNA fragmentation, particularly in samples with high DFI. However, this improvement does not necessarily guarantee better clinical outcomes in IVF. Baseline sperm DNA fragmentation index (DFI) may still act as an independent prognostic factor influencing IVF success, regardless of the sperm preparation method used.

## INTRODUCTION

Infertility affects approximately 15% of couples worldwide, with male factors contributing to nearly half of these cases. While conventional semen analysis remains a cornerstone in evaluating male fertility, it often falls short in predicting successful fertilization and pregnancy outcomes. Notably, sperm DNA fragmentation (SDF) has emerged as a critical parameter, with elevated SDF levels associated with impaired embryo development, reduced blastocyst quality, and increased miscarriage rates (Conti *et al.*, 2024; Agarwal *et al.*, 2020).

Traditional sperm preparation techniques, such as density gradient centrifugation (DGC), are widely employed to isolate motile and morphologically normal spermatozoa. However, these methods involve multiple centrifugation steps and exposure to colloidal silica media, which can generate reactive oxygen species (ROS), potentially compromising sperm membrane integrity and inducing DNA fragmentation (Muratori *et al.*, 2019; Agarwal *et al.*, 2020). Consequently, there is a growing interest in alternative, less invasive sperm selection methods that can minimize DNA damage and enhance assisted reproductive technology (ART) outcomes.

Microfluidic sperm sorting (MC) has emerged as a promising technique that mimics the natural selection processes of the female reproductive tract. Utilizing laminar flow within microchannels, microfluidic devices facilitate the selection of highly motile sperm with intact DNA by leveraging mechanisms such as rheotaxis and chemotaxis. These devices offer several advantages over conventional methods, including reduced processing time, minimal handling, and decreased operator dependency.

The concept of microfluidics was first introduced in the early 1990s in the context of analytical chemistry and biomedical diagnostics. Its application to reproductive medicine began to gain traction in the early 2000s when researchers recognized that the precise fluid dynamics of microchannels could be harnessed to mimic the physiological environment of the female reproductive tract. By the 2010s, commercially available sperm-sorting chips such as ZyMōt™, FERTILE™, and FERTILE PLUS™ began to appear, offering ART centers a more controlled, physiologically relevant method for sperm preparation. These platforms aimed to reduce oxidative stress exposure and preserve DNA integrity in sperm selected for ICSI (Quinn *et al.*, 2018; Anbari *et al.*, 2021; Yildiz & Yuksel, 2019).

Several studies have investigated the efficacy of MC in improving ART outcomes. For instance, a retrospective cohort study by Pujol et al. demonstrated that the use of a microfluidic sperm sorting device significantly reduced double-stranded DNA fragmentation by 46% compared to the swim-up method (Pujol *et al*., 2022). Similarly, Vahidi et al. reported improvements in sperm morphology, motility, and DNA integrity when using microfluidic sorting techniques (Vahidi *et al.*, 2025).

Clinical outcomes have also been evaluated in studies comparing MC to traditional methods. A study by Banti et al. found that the use of the FERTILE PLUS™ microfluidic sperm sorting chip resulted in higher blastocyst formation rates (76% *vs*. 56%) and euploidy rates (40% *vs*. 20%) compared to DGC (Banti *et al*., 2024). Although the increase in fertilization rates was not statistically significant, the findings suggest potential benefits of microfluidic sorting in enhancing embryo quality.

Despite these promising results, some studies have reported marginal improvements without statistical significance. A meta-analysis concluded that while MC demonstrates slight positive outcomes compared to standard techniques, the differences were not statistically significant across analyzed parameters (Ferreira Aderaldo *et al.*, 2023). The authors emphasized the need for larger, multicenter studies with standardized protocols to validate the clinical benefits of MC.

Additional investigations have also echoed these findings. Ozcan et al., in a comparative study involving 181 infertile males, reported a higher clinical pregnancy rate in the MC group (49.5%) compared to the DGC group (40%), but the difference was not statistically significant (*p*=0.2) (Ozcan *et al*., 2021). Similarly, Quinn et al. found no significant improvement in embryo quality or clinical pregnancy rates between MC and DGC in ICSI cycles (Quinn *et al*., 2022). These findings suggest that while MC technologies hold promise in improving sperm DNA integrity, their translation into enhanced clinical outcomes remains inconsistent.

In light of current evidence, MC presents a compelling alternative to conventional sperm preparation methods, particularly in cases with high SDF levels. However, further research is necessary to establish its efficacy in improving clinical outcomes consistently. This study aims to compare sperm quality and ART outcomes between MC and DGC, providing insights into the practical applications of MC technology in routine IVF procedures. Moreover, unlike most previous studies, our research was designed to extend follow-up beyond fertilization and early embryo development, with particular emphasis on evaluating live birth outcomes as the ultimate endpoint of assisted reproduction success.

## MATERIALS AND METHODS

### Inclusion and Exclusion Criteria

This randomized controlled study was conducted on 119 couples who underwent IVF cycles at the Center of IVF and Tissue engineering - Hanoi Medical University Hospital, from March 2023 to March 2024, following ethical approval by the Hanoi Medical University Institutional Ethical Review Board (HMU IRB). All participants used autologous oocytes and ejaculated sperm, with no use of donor gametes. On the male side, exclusion criteria included patients requiring testicular sperm extraction or those diagnosed with oligoasthenozoospermia (OA) (WHO, 2021). On the female side, couples were excluded if the female partner was classified as having a poor ovarian prognosis according to the POSEIDON 2016 criteria. This includes women with reduced ovarian reserve, defined by anti-Müllerian hormone (AMH) levels <1.2 ng/mL or antral follicle count (AFC) <5, or those with normal reserve but a history of poor ovarian response (≤9 oocytes retrieved) in previous cycles. Preimplantation genetic testing (PGT) cycles were also excluded from the study.

### Study Design

Couples who met inclusion criteria were randomized into two groups: the intervention group (MC group), in which semen was processed using a microfluidic sperm sorting device and the control group (DGC group), in which sperm was prepared using traditional density gradient centrifugation.

Before patient recruitment began, a list of 120 random numbers was generated using the RAND() function in Microsoft Excel. The list was then sorted in ascending order. The first 60 positions were allocated to the intervention group (MC), and the remaining 60 to the control group (DGC). During the study, eligible patients were consecutively enrolled according to the actual order of recruitment and assigned to the corresponding group based on the pre-determined randomization list. However, one couple in the control group withdrew from treatment before oocyte retrieval, resulting in a final sample size of 60 couples in the MC group and 59 in the DGC group.

Both groups followed the same controlled ovarian stimulation protocol and laboratory workflow for IVF treatment. On the day of oocyte retrieval, semen samples were processed according to the assigned technique and used for intracytoplasmic sperm injection (ICSI). Embryologists performing ICSI and embryo assessment were blinded to the group allocation to minimize bias.

The primary outcomes evaluated were the sperm DNA fragmentation index (DFI) and the live birth rate. Secondary outcomes included sperm quality after preparation (motility and morphology), fertilization rate, number and quality of embryos on day 2, and pregnancy outcomes, including biochemical, clinical, and ongoing pregnancies.

### Sperm Preparation Protocols

#### a. Density Gradient Centrifugation

Semen samples in the control group were processed using a discontinuous density gradient system. Briefly, 1 mL of 90% gradient medium (SpermGrad™, Vitrolife, Sweden) was layered beneath 1 mL of 45% gradient medium in a centrifuge tube. Then, 1 mL of liquefied semen was gently added on top. The sample was centrifuged at 345 × g for 8 minutes. The resulting pellet was washed with 4 mL of sperm washing medium (SpermRinse™, Vitrolife, Sweden) and centrifuged again at the same speed for 5 minutes. The final pellet was resuspended in 0.3 - 0.5 mL of washing medium.

#### b. Microfluidic technique

For the intervention group, semen was processed using the ZyMōt Multi Sperm Separation Device (850 µL; DxNow Inc., USA). A total of 850 µL of liquefied semen was first slowly loaded into the inlet port. Subsequently, 750 µL of washing medium was added to the device, including 50 µL to prime the outlet port and 700 µL to cover the membrane surface. The device was incubated for 30 minutes at 37°C. The sorted sperm was collected from the outlet port, with a final volume of up to 500 µL.

### Sperm DNA fragmentation assessment (SCSA)

Sperm DNA fragmentation was assessed using the PhacoSperm® DNA Fragmentation Kit, which is based on the sperm chromatin structure assay (SCSA). This assay relies on the differential fluorescence of acridine orange (AO) when bound to double-stranded DNA (green emission) versus single-stranded DNA (red emission) under excitation by blue laser light. After acid-induced denaturation, fragmented DNA becomes single-stranded, while intact chromatin remains double-stranded. Flow cytometry was performed to determine the DNA fragmentation index (DFI) by analyzing fluorescence signals from 5,000 spermatozoa per sample. A DFI greater than 15% was considered abnormal.

### Ovarian stimulation, oocyte retrieval, and ICSI

#### a. Ovarian stimulation

Controlled ovarian stimulation was initiated with recombinant follicle-stimulating hormone (rFSH; Gonal-F^®^, Merck Serono, Italy) on cycle day 2. The dose was adjusted according to individual ovarian reserve. A GnRH antagonist (Cetrotide^®^, 250 µg; Merck Serono, Germany) was administered from day 5 or 6 until the ovulation trigger. When at least two follicles reached ≥18 mm in diameter, final oocyte maturation was induced using Ovitrelle® (250 µg; Merck Serono).

#### b. Oocyte retrieval and ICSI

Oocyte retrieval was performed 34-36 hours after hCG administration via transvaginal ultrasound-guided aspiration under sedation. Following a 2-hour incubation period, cumulus cells were removed using hyaluronidase (HYASE-10X™, Vitrolife^®^, Sweden). Sperm prepared by either density gradient centrifugation (DGC) or microfluidic sorting was injected into mature oocytes using intracytoplasmic sperm injection (ICSI). Fertilization was confirmed 17-20 hours later by the presence of two pronuclei. Embryos were cultured in Continuous Single Culture^®^-NX Complete medium (FUJIFILM Irvine Scientific, USA) in a tri-gas incubator (5% CO₂, 5% O₂, 90% N₂) at 37°C.

#### c. Embryo quality assessment

Embryo quality was assessed on day 2 in accordance with the 2011 Istanbul consensus. Good-quality embryos were defined as those containing 4-6 evenly sized blastomeres with less than 10% fragmentation. Moderate-quality embryos exhibited 10-25% fragmentation, while poor-quality embryos showed uneven blastomeres or greater than 25% fragmentation.

### Embryo transfer and pregnancy outcomes

#### a. Endometrial preparation

Estrogen supplementation with estradiol valerate (6-8 mg/day) was initiated on cycle day 2. Endometrial thickness was assessed by transvaginal ultrasound on day 10. If the thickness was less than 8 mm, the dose was increased to 12-16 mg/day and reassessed. Progesterone was initiated when the endometrial thickness reached ≥8 mm, in the absence of intrauterine fluid, and when a trilaminar endometrial pattern was observed. Embryo transfer cycles were canceled if the endometrial thickness remained <8 mm or exceeded 14 mm, if intrauterine fluid was present, or if the endometrial pattern appeared diffusely hyperechogenic.

#### b. Embryo transfer and pregnancy assessment

Embryo transfer was performed on day 3, day 4, or day 5. Serum β-hCG levels were measured 10 days after day-5 embryo transfer or 12 days after day-3 embryo transfer. Biochemical pregnancy was defined as a serum β-hCG level greater than 25 IU/L. Clinical pregnancy was confirmed by the presence of a gestational sac and fetal heartbeat at 4 weeks. Ongoing pregnancy was confirmed by ultrasound at 12 weeks of gestation. Live birth was defined as the complete expulsion or extraction of a fetus from the mother, showing any sign of life, regardless of gestational age.

### Statistical analysis

Data analysis was performed using SPSS version 20.0 (IBM Corp., Armonk, NY, USA). Continuous variables were expressed as mean±standard deviation (SD), while categorical variables were presented as frequency and percentage. Group comparisons for categorical variables were performed using the Chi-square test and the Fisher exact test in the case of expected frequency in any cell being less than 5. For continuous variables, the Student’s T-test was used to compare normally distributed data, and the Mann-Whitney U test was applied for non-normally distributed data. A two-tailed *p*-value less than 0.05 was considered statistically significant.

### Ethical considerations

Patient information was anonymized, kept confidential, and used solely for research purposes. The study was conducted only after obtaining written informed consent from all participants. This comparative trial was approved by the leadership of the Center of IVF and Tissue engineering - Hanoi Medical University Hospital, and received ethical approval from the Hanoi Medical University Institutional Ethical Review Board (IBR-VN01.001 / IRB00003121 / FWA00004148)

**Clinical Trial Registration:** This study was registered at ClinicalTrials.gov with the identifier NCT07004309.

## RESULTS

A total of 119 couples completed the study and were included in the final analysis, with 60 in the MC group and 59 in the DGC group.

### Baseline characteristics of patients between the DGC and MC groups

Compared to the DGC group, the MC group had significantly younger female (29.73±3.77 *vs*. 31.47±4.16 years, *p=*0.018) and male participants (32.27±3.29 *vs*. 34.81±4.59 years, *p=*0.001). Moreover, anti-Müllerian hormone (AMH) levels were significantly higher in the MC group than in the DGC group (5.63±4.09 *vs*. 3.79±2.55 ng/mL, *p=*0.004), indicating a better ovarian reserve in this cohort (Table 1).

**Table 1:** Baseline demographic and clinical characteristics of participants in the DGC and MC groups Values are presented as mean±standard deviation for continuous variables and as number (percentage) for categorical variables. Significant differences were observed in female age, male age, and AMH levels (*p*<0.05). No statistically significant differences were found between groups in BMI, infertility type, duration of infertility, AFC, or causes of infertility. Sample size: DGC group (n=59), MC group (n=60). Abbreviations: DGC=Density Gradient Centrifugation, MC=Microfluidic

Parameter		DGC group (n=59)	MC group (n=60)	*p*-value	Unit
Female age		31.47±4.16	29.73±3.77	0.018	years
Male age		34.81±4.59	32.27±3.29	0.001	years
Female BMI		22.11±2.51	22.12±2.97	0.987	kg/m^2^
Male BMI		23.26±2.37	23.75±3.44	0.368	kg/m^2^
Infertility type (Primary)		33.9%	46.7%	0.156	%
Duration of infertility		2.77±2.39	3.10±2.40	0.452	years
AMH		3.79±2.55	5.63±4.09	**0.004**	ng/mL
AFC		17.23±9.53	18.45±9.15	0.477	N
Infertility cause	Tubal factors (34)	15/59 (25.4%)	19/60 (31.7%)	0.451	
	Endometriosis (5)	3/59 (5.1%)	2/60 (3.3%)	0.679	
	Uterine abnormalities (3)	2/59 (3.4%)	1/60 (1.7%)	0.619	
	Endocrine/genetic disorders (9)	4/59 (6.8%)	5/60 (8.3%)	1.000	
	PCOS (63)	35/59 (59.3%)	28/60 (46.7%)	0.167	
	Other causes (9)	2/59 (3.4%)	7/60 (11.7%)	0.163	

### Semen parameters before and after sperm preparation

Both DGC and MC methods significantly improved semen parameters, including progressive motility, viability, and normal morphology (all *p*<0.0001). However, a key advantage of the microfluidic technique lies in its superior capacity to reduce the sperm DNA fragmentation index (DFI) (Table 2). Post-preparation DFI was significantly lower in the MC group (0.43±0.38%) compared to the DGC group (2.47±5.08%, *p*=0.0024), particularly in samples with high baseline DFI (≥15%), where the MC group achieved a marked reduction (*p*<0.0001). In samples with low baseline DFI (<15%), both methods were effective; however, the MC group still demonstrated a significantly lower final DFI (*p*=0.0001) (Table 2).

**Table 2:** Comparison of semen parameters before and after preparation using Density Gradient Centrifugation (DGC) and Microfluidics (MC) Values are presented as mean±standard deviation. Within-group comparisons (p₁₂, p₃₄) and between-group comparisons (p₁₃, p₂₄) are shown. Both methods significantly improved motility, viability, morphology, and DFI (*p*<0.0001). The MC group showed lower post-preparation DFI and higher motile sperm recovery rate. Abbreviations: DFI=DNA Fragmentation Index

Parameter	Before preparation (DGC group) (1)	After preparation (DGC group) (2)	Before preparation (MC group) (3)	After preparation (MC group) (4)	*p*
Sperm concentration (10^6^/mL)	105.15±58.35	39.19±30.77	81.06±47.79	33.35±33.72	p₁₂<0.0001 p₃₄<0.0001 p₁₃=0.015 p₂₄=0.326
Progressive motility (%)	49.57±19.33	90.12±5.51	39.60±18.05	84.22±11.03	p₁₂<0.0001 p₃₄<0.0001 p₁₃=0.004 p₂₄=0.0004
Viability (%)	84.25±8.88	96.95±3.71	81.97±8.57	97.45±2.97	p₁₂<0.0001 p₃₄<0.0001 p₁₃=0.155 p₂₄=0.900
Normal morphology (%)	3.41±1.26	7.80±2.91	3.23±1.40	8.52±3.33	p₁₂<0.0001 p₃₄<0.0001 p₁₃=0.478 p₂₄=0.211
DNA Fragmentation Index (DFI,%)	10.86±7.99	2.47±5.08	12.02±9.46	0.43±0.38	p₁₂<0.0001 p₃₄<0.0001 p₁₃=0.472 p₂₄=0.0024
DFI<15%	n=46 7.22±2.95 (2.33 - 13.88)	n=46 0.99±1.04 (0.09 - 5.51)	n=45 7.79±3.73 (1.57 - 4.75)	n=45 0.33±0.28 (0.02 - 1.08)	p₁₂<0.0001 p₃₄<0.0001 p₁₃=0.418 p₂₄=0.0001
DFI ≥15%	n=13 23.74±6.73 (15.69 - 41.12)	n=13 6.10±7.74 (0.93 - 26.70)	n=15 24.71±10.19 (16.53 -52.42)	n=15 0.73±0.48 (0.21 - 1.83)	p₁₂<0.0001 p₃₄<0.0001 p₁₃=0.774 p₂₄<0.0001
Sperm recovery rate		20.59±14.28		24.60±20.62	0.220
Motile sperm recovery rate		30.59±2.01		44.41±3.95	<0.0001

Furthermore, the MC group exhibited a significantly higher motile sperm recovery rate (44.41±3.95% *vs*. 30.59±2.01%, *p*<0.0001), suggesting improved selection of functionally competent sperm. In contrast, total sperm recovery and post-preparation morphology were comparable between the two groups (Table 2).

**Figure 1:**
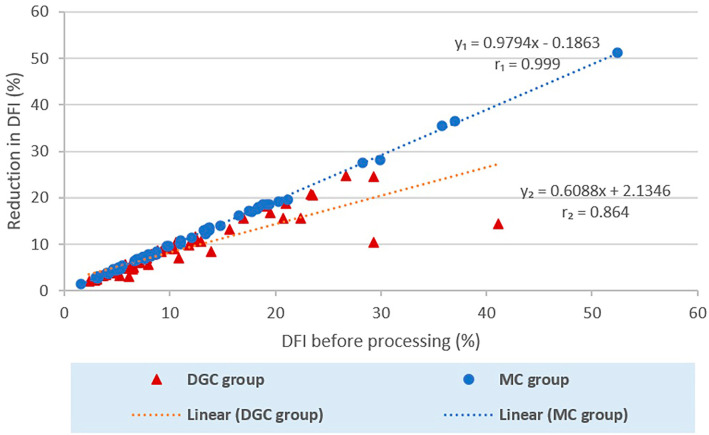
Correlation between initial DNA Fragmentation Index (DFI) and reduction in DFI after sperm processing using two different methods. The graph illustrates the relationship between DFI before processing and the percentage reduction in DFI in two groups: the DGC group (red triangles) and the MC group (blue circles). Linear regression lines were fitted for each group.

Linear regression analysis revealed a strong correlation between preand post-processing DFI in the MC group (r₁=0.999), indicating a proportional reduction in DFI across the full range of initial values (Figure 1). Notably, samples with higher pre-processing DFI experienced greater reductions, highlighting the clear advantage of microfluidic technology in improving DNA integrity, especially in cases with severe damage. By contrast, the DGC group showed a weaker and more variable reduction (r₂=0.864), suggesting that DFI reduction was more dependent on other factors, making the outcomes less predictable (Figure 1).

### Laboratory and clinical IVF outcomes

The number of oocytes retrieved was significantly higher in the Microfluidics (MC) group compared to the DGC group (19.63±8.80 *vs*. 16.27±7.28, *p*=0.025). However, no significant differences were observed between the groups in mature oocyte rate (68.89±19.36% *vs*. 68.00±17.64%, *p*=0.792), fertilization rate (92.67±9.45% *vs*. 93.85±9.12%, *p*=0.490), Day 2 embryo formation rate (98.34±3.75% *vs*. 98.70±3.75%, *p*=0.594), or good-quality Day 2 embryo rate (71.08±24.45% *vs*. 71.61±22.95%, *p*=0.903) (Table 3).

**Table 3:** Comparison of laboratory outcomes between the DGC and MC groups Values are presented as mean±standard deviation. The MC group showed a significantly higher number of oocytes retrieved (*p*=0.025). No significant differences were observed in mature oocyte rate, fertilization rate, Day 2 embryo formation rate, or good-quality Day 2 embryo rate.

Outcome	DGC group (n=59)	MC group (n=60)	p-value	Unit
Number of oocytes retrieved	16.27±7.28	19.63±8.80	0.025	n
Mature oocyte rate	68.00±17.64	68.89±19.36	0.792	%
Fertilization rate	93.85±9.12	92.67±9.45	0.490	%
Day 2 embryo formation rate	98.70±3.75	98.34±3.75	0.594	%
Good-quality Day 2 embryo rate	71.61±22.95	71.08±24.45	0.903	%

Embryo transfer and pregnancy outcomes are shown in Table 4. Pregnancy rates following Day 5, Day 4, and cleavage-stage transfers were not significantly different. Total pregnancy rate (75.5% *vs*. 72.2%, *p=*0.704), clinical pregnancy rate (67.4% *vs*. 64.8%, *p=*0.786), ongoing pregnancy rate (63.3% *vs*. 59.3%, *p=*0.676), and live birth rate (63.3% *vs*. 59.3%, *p=*0.676) were comparable between the DGC and MC groups (Table 4).

**Table 4:** Comparison of embryo transfer outcomes and pregnancy results between the DGC and Microfluidics (MC) groups Values are presented as number of cases (percentage). No significant differences were observed between groups in overall pregnancy rates, clinical pregnancy, ongoing pregnancy, or live birth rates.

Outcome	DGC group (n=49)	MC group (n=54)	*p*-value
Day-5 embryo transfer pregnancy rate	29/35 (82.9%)	27/32 (84.4%)	0.867
Day-4 embryo transfer pregnancy rate	1/2 (50.0%)	7/10 (70.0%)	1.000
Cleavage-stage embryo transfer pregnancy rate	7/12 (58.3%)	5/12 (41.67%)	0.414
Pregnancy rate (total)	75.5% (37/49)	72.2% (39/54)	0.704
Clinical pregnancy rate (total)	67.4% (33/49)	64.8% (35/54)	0.786
Ongoing pregnancy rate (total)	63.3% (31/49)	59.3% (32/54)	0.676
Live birth rate (total)	63.3% (31/49)	59.3% (32/54)	0.676

Table 5 presents pregnancy outcomes according to baseline DFI levels in both the DGC and MC groups. Within each group, patients with high DFI (≥15%) showed slightly lower pregnancy-related outcomes compared to those with low DFI (<15%); however, none of the differences reached statistical significance.

**Table 5:** Pregnancy outcomes in DGC and Microfluidics (MC) groups stratified by baseline DNA fragmentation index (DFI)Values are presented as number of cases (percentage). Outcomes are compared between patients with low (DFI < 15%) and high (DFI ≥ 15%) DNA fragmentation in each group. No statistically significant differences were observed in pregnancy, clinical pregnancy, ongoing pregnancy, or live birth rates between subgroups.

Outcome	DGC group (n=49)		MC group (n=54)		*p*-value
	DFI < 15% (n=37) (1)	DFI ≥15% (n=12) (2)	DFI < 15% (n=40) (3)	DFI ≥15% (n=14) (4)	
Pregnancy rate	29 (78.4%)	8 (66.7%)	30 (75.0%)	9 (64.3%)	p₁-₂=0.454 p₃-₄=0.441 p₂-₄=1.000
Clinical pregnancy rate	25 (67.6%)	8 (66.7%)	26 (65.0%)	9 (64.3%)	p₁-₂=1.000 p₃-₄=0.961 p₂-₄=1.000
Ongoing pregnancy rate	24 (64.9%)	7 (58.3%)	25 (62.5%)	7 (50.0%)	p₁-₂=0.683 p₃-₄=0.412 p₂-₄=0.712
Live birth rate	24 (64.9%)	7 (58.3%)	25 (62.5%)	7 (50.0%)	p₁-₂=0.683 p₃-₄=0.412 p₂-₄=0.712

In the DGC group, live birth rates were 64.9% for DFI <15% and 58.3% for DFI ≥15% (p=0.683). Similarly, in the MC group, live birth rates were 62.5% *vs*. 50.0% for low *vs*. high DFI (*p*=0.412). Comparisons between the high-DFI subgroups of both groups also revealed no significant differences (*p*=0.712). These results suggest that both preparation methods may mitigate the adverse impact of elevated DFI on pregnancy outcomes (Table 5).

## DISCUSSION

This randomized controlled trial aimed to compare the effectiveness of MC and DGC for sperm preparation in IVF cycles. The primary outcomes included live birth rates and sperm DNA fragmentation index (DFI), while secondary outcomes encompassed sperm quality, fertilization rates, embryo quality, and overall pregnancy outcomes. The findings provide valuable insights into the relative advantages and limitations of these two sperm preparation methods.

### Baseline characteristics and randomization

In this study, we successfully recruited 119 couples and randomly assigned them into two groups using a random sequence. Despite efforts to minimize confounding factors by excluding women with poor ovarian response according to the POSEIDON criteria and ensuring that the causes of infertility were comparable between the two groups, we still observed some significant differences. Specifically, the intervention group (MC) had a younger average age for both female (29.73±3.77 *vs*. 31.47±4.16 years, *p*=0.018) and male partners (32.27±3.29 *vs*. 34.81±4.59 years, *p*=0.001), along with a higher ovarian reserve (AMH 5.63±4.02 *vs*. 3.79±2.55 ng/mL, *p*=0.004) compared to the control group (DGC). These differences could potentially introduce a favorable bias for the MC group, as younger couples with higher ovarian reserves generally have better reproductive outcomes. However, previous studies have indicated that the age thresholds that significantly impact IVF success are 35 years for women and 40 years for men (Vitagliano *et al.*, 2023; Gao *et al.*, 2024; Lu *et al.*, 2023). Therefore, despite the age differences between the two groups, the fact that the average ages in both groups were below these critical thresholds suggests that this disparity is unlikely to significantly influence the overall study outcomes.

### Comparison of DFI Reduction Efficiency between Microfluidics and DGC Methods

Numerous studies have confirmed that MC is more effective in reducing sperm DFI compared to traditional DGC, especially in samples with high DFI (Anbari *et al.*, 2021; Quinn *et al.*, 2018; Keskin *et al.*, 2022). Our study not only supports this finding but also highlights the consistency of the technique: the reduction in DFI in the MC group was not only more pronounced but also showed a nearly perfect linear correlation between preand post-processing DFI (r=0.999), indicating a stable reduction across the entire DFI spectrum regardless of baseline DNA damage.

In contrast, DGC showed less consistent DFI reduction, particularly in samples with high DFI. The lower correlation coefficient (r=0.864) suggests variability in DFI reduction efficacy. This may be due to steps in the DGC process, such as centrifugation and exposure to resin particles, which can cause mechanical and oxidative stress on sperm. Several studies have also indicated that DGC may not be suitable for all sperm samples, especially those with poor quality (OAT), from older men, or with high oxidative stress-commonly seen in men who smoke, are exposed to toxins, or have unhealthy lifestyles (Conti *et al.*, 2024; Muratori *et al.*, 2019). Although our study excluded OA samples and limited male age to under 40 to minimize confounding, factors such as smoking and lifestyle are difficult to control completely and may affect DGC efficiency. Therefore, for semen samples with DFI ≥15% or in men with a history of smoking or unhealthy habits, it may be advisable to consider Microfluidics as the initial sperm selection method for ICSI.

### Microfluidics filtration mechanism and benefits for sperm and IVF outcomes

Microfluidics mimics the physiological environment of the female reproductive tract, allowing sperm to actively migrate through microchannels without centrifugation or exposure to chemicals. This significantly reduces mechanical and oxidative stress-two major causes of sperm DNA damage. The technology also allows for effective selection of highly motile, morphologically normal sperm while eliminating dead or DNA-damaged sperm. These benefits are particularly evident in samples with high DFI (Agarwal *et al.*, 2020).

Several recent studies have reported that MC may improve IVF outcomes in certain selected patient populations, such as increasing fertilization rates in recurrent IVF failure cases (Yildiz & Yuksel, 2019), enhancing blastocyst formation rates (Ozaltin *et al.*, 2023), or improving blastocyst quality and euploidy rates in patients with astheno-teratozoospermia (Guler *et al.*, 2021). However, a recent meta-analysis found no statistically significant difference in clinical pregnancy rates when comparing MC with conventional sperm preparation methods. This suggests that while MC may enhance sperm quality, it does not necessarily translate into improved clinical IVF outcomes (Ferreira Aderaldo *et al.*, 2023).

In our study, we intentionally excluded low-quality and low-count sperm samples due to concerns about sperm recovery for ICSI-this exclusion criterion effectively defined a selected patient population. When comparing clinical outcomes between the two groups, no significant differences were observed in fertilization rates, embryo formation, day-2 embryo quality, pregnancy rates, clinical pregnancy, ongoing pregnancy, or live birth rates. The only notable difference was a higher number of oocytes retrieved in the MC group, which is consistent with the younger age and better ovarian reserve of the women in that group.

Even when further stratifying outcomes based on baseline DFI levels (≥15% *vs*. <15%), no significant differences were found in post-transfer success rates. This indicates that although MC is effective at reducing DFI-especially in high-DFI samples-it is not sufficient on its own to significantly improve IVF outcomes. Moreover, in both DGC and Microfluidics groups, clinical pregnancy rates tended to be lower in patients with DFI ≥15%, suggesting that baseline DFI may remain an independent prognostic factor affecting IVF outcomes, regardless of the sperm preparation method used.

### Limitations

This study has some methodological limitations that should be acknowledged. First, the sample size was not calculated a priori, as the study was designed as an exploratory trial. Second, it was conducted at a single center, which may limit the generalizability of the findings to other clinical settings. While embryologists performing ICSI and embryo assessment were blinded to group allocation, sperm preparation was not blinded, which could introduce a degree of performance bias. Moreover, the intervention group had slightly more favorable baseline characteristics, such as younger female age and higher AMH levels, which may have influenced outcomes. Lastly, neonatal outcomes were not evaluated, as the study focused primarily on live birth as the final endpoint.

## CONCLUSION

Microfluidics is an effective method for reducing sperm DNA fragmentation, particularly in samples with high DFI. However, this improvement does not necessarily guarantee better clinical outcomes in IVF. Baseline sperm DNA fragmentation index (DFI) may still act as an independent prognostic factor influencing IVF success, regardless of the sperm preparation method used.
